# Supplementary material for: Low profile high gain RHCP antenna for L-Band and S-Band using rectangular ring metasurface with backlobe suppression
Source: PLoS One. 2024 Feb 8;19(2):e0297957. doi: 10.1371/journal.pone.0297957 (PMC10852314; doi:10.1371/journal.pone.0297957)
Supplement: Attachment — Submitted filename: Response to Reviewer.docx [file pone.0297957.s001.docx]

| **Reviewer 1**  I loved this work and I feel it is very good. I hope these comments will help you improve this work after a major revision | **Comments** | **Action** |
| --- | --- | --- |
|  | 1-Can you summarize in a few sentences what all this work is about at the end of the introduction? | Last Para of Introduction, Line 47-52.  “In summary, this work introduces a novel low-profile metasurface antenna designed for the L-band in satellite communication. Utilizing commonly available materials and a simplified configuration, the proposed antenna aims to reduce size, enhance gain and bandwidth, and maintain system reliability. Addressing a notable gap in existing literature, this research contributes to the exploration of metasurface applications in satellite communication, particularly in the less-explored L-band frequency range, using commonly available FR 4 substrate.” |
|  | 2-The abstract needs more interest and rewriting of some paragraphs. | “In this reported work a single feed, miniaturized, dual layer, miniaturized, and low profile antenna is presented for 1.575GHz frequency band. The proposed antenna offers high gain, lower noise bandwidth, with better sensitivity and range. The ground choke technique is used for back lobe suppression. The prototype is fabricated on FR 4 substrate using manual fabrication technique. This offers an inexpensive and readily available fabrication. The antenna comprises of two layers, containing a patch radiator and a Metasurface layer with 3x3 rectangular ring resonators. The layers are separated using foam with a 12mm width. The proposed prototype is radiating at 1.575GHz and 2.33GHz with an overall dimension of 85.6 x 68.4 x 15.204 mm. The developed antenna provides a gain of 5.9 dBi. The simulated results are verified using VNA and anechoic chamber testing. Moreover, the developed antenna has been successfully tested for L-Band Satellite communication in real time scenario without any LNA. Higher Gain due to Metasurface increase the efficiency of the system. The promising results indicate the aptness of the developed antenna for real-world applications of L-Band and S-Band.” |
|  | 3-There are still some aspects that can be improved (for grammar and punctuation). Improve the technical writing of your paper, where there are several grammatical errors and spelling I think they need to be checked out. | Done |
|  | 4-The conclusion needs more effort to elaborate on the achieved results with respect to future work, | Done |
|  | 5-There are still some aspects regarding the obtained results discussions that are missing. Can you please address your achievements well? | Done |
|  | 6-The practical part is very important, therefore, I’m asking about the radiation pattern measurements, | Done Fig 10 |
|  | 7-Why this design geometry, explain the design methodology and specify the novelty of your antenna structure, | Done in 3^rd^ & 2^nd^ last para of Design & Analysis Line No 82-93  “Functionality of Metasurface layer can be understood through superstrate or resonant cavity Antenna [27]. By appropriately designing the scaterrers and controlling the phase and amplitude of wave it's possible to enhance the amplitude of the waves in the desired direction, effectively increasing the gain of the antenna in that direction. Left- handed Metametrial (LH-MTMs) or Double negative (DNG) materials as well as Electronic bandgap structure (EBG) also enhance gain and directivity when primary source antenna is paired with them [28-30]. In [27] double closed ring resonator (DCR) as reflective metasurface unit cell was investigated and was found that at 2.2 GHz and 2.9GHz it shows unusual properties such as permeability close to zero. Retrieved effective parameters shows DCR lies in Mu Near Zero (MNZ) & Epsilon Negative medium (ENG) region at 2.2 and 2.9GHz frequency thus enhancing gain.  For our desired frequency i.e. 1.575GHz we designed the single closed ring resonator with bigger dimensions as shown in Fig 2(a). Only unit cell was also designed separately through floquet port of CST MW. It was concluded that metasurface in subject case is collimating the wave nature thus increasing directivity and gain. Further study on retrieved effective parameters is in progress.” |
|  | 8-Future work is an important part of the conclusion. | 1-Study of Retrieved effective parameters (2^nd^ last para of Design & Analysis para Line No 93)  “Further study on retrieved effective parameters is in progress.”  2-Further designing on dual band (2^nd^ Para of Results & Discussion Line No 138-139)  “Antenna is circularly polarized at 1.575GHz however it is linearly polarized at 2.33GHz at the time. But it can be further optimized to make it circularly polarized at 2.33GHz as well.”  3-Conclusion  “Study of retrieved effective parameters, further optimization of antenna for S-Band to increase multi-functionality is the proposed future work. Further size reduction and exploring the design on different substrate are also termed as future activities.” |
|  | 9-The results are still not matured well. Can you clearly mention what your objectives clearly for each result you got during the discussion process? | Results & Discussion 1^st^ para Line No 122-124  “In order to qualify antenna for satellite communication reception its reflection coefficients, polarization and gain was checked.” |
|  | 10- There are several relative works, I wish to discuss, based on adding such structures to the antenna. Can you please suggest your enhancements and advancements (if you would) over such as the following | Delineated in table 2. |
| **Reviewer 2**  The authors presented their work, "Low Profile High Gain RHCP Antenna for L- Band and S-Band using Rectangular Ring  Metasurface with Backlobe suppression", which seems to be quite interesting and important from a research point of view considering its outcomes. However, some technical modifications are required before considering revising it. | First of all, the authors are required to highlight the need for the proposed metasurface antenna design by explaining its pros and cons over the ones reported in the existing literature. So, a brief inclusion of it is required in the manuscript. | Last Para of Results & Discussion Line No 184 -189  “The presented prototype has an edge over its predecessor in size reduction, gain enhancement and simplicity. As previously reported, work for same frequency i.e., 1.575GHz was fabricated using customized Rogers substrate. In [26] 2-layer design was presented with 9dBi gain but with large dimensions. In [31] 3-layer design was presented adding complexity in manufacturing process and 5dBi gain. This paper presents a design which gives more than 50% size reduction over [31] and better gain than [32]. Moreover, it has added advantage of easy fabrication on readily available substrate. A comparison is delineated in Table 2.” |
|  | The analysis of the metasurface unit cells is missing. On what basis it is working in the proposed antenna design needs to be explained. | Done in 2^nd^ last para of Design & Analysis Line No 90-94  “For our desired frequency i.e. 1.575GHz we designed the single closed ring resonator with bigger dimensions as shown in Fig 2(a). Only unit cell was also designed separately through floquet port of CST MW. It was concluded that metasurface in subject case is collimating the wave nature thus increasing directivity and gain. Further study on retrieved effective parameters is in progress.” |
|  | Considering the attainment of circular polarization (CP) outcomes, it is significant to add the analysis in terms of electric field distribution, surface current distribution, radiation pattern, etc. In the radiation pattern, LHCP and RHCP components should be shown clearly. | Electric Field Distribution Fig 11(a) (New Figure)  Surface Current Distribution Fig 11(b) (New Figure)  Radiation Pattern Fig 10 (Replotted, already presented work on Origin software)  LHCP & RHCP Component Fig 9 (b) & (c) (Already Existed) |
|  | The authors should try to plot the obtained results by using the plotting software. | Anechoic chamber results were replotted and are presented in Fig 10. Results are plotted using Origin software. Fig 10a presents polar plots whereas Fig 10b presents normalized plots in Cartesian. |
|  | Finally, a comparison table must be prepared, highlighting the performance aspects, mainly CP characteristics, of the proposed metasurface antenna compared to others | Table 2 |

| **Related Works Suggested by Reviewer 1** | **Paper** | **Remarks** |
| --- | --- | --- |
| <https://doi.org/10.1016/j.aeue.2023.154652> | Photonic controlled metasurface for intelligent antenna beam steering applications including 6G mobile communication systems | This paper presents an active antenna with Metasurafce as superstrate for Gain enhancement. Active layer includes diode for beamforming in desired direction.  Referred [28] |
| <https://doi.org/10.1002/mop.33761> | Reconfigurable CRLH-inspired antenna based on Hilbert curve EBG structure for modern wireless systems | 17 unit cells of a composite right/left‐hand (CRLH) structure with an electromagnetic band gap of Hilbert inclusions is presented wich employs direct amplitude modulation technique  Referred [30] |
| <https://doi.org/10.36244/ICJ.2023.2.1> | Metasurface Antenna Circuitry for 5G Communication Networks | A 12 unit Metasurface around the patch is presented for wave suppression.  Referred [29] |
| <https://doi.org/10.32620/reks.2023.1.06> | Reconfigurable composite right/left-handed transmission line antenna based Hilbert/Minkowski stepped impedance resonator for wireless applications | Analysis and design of a via free metamaterial antenna based on a composite right/left-handed transmission line (CRLH-TL) structure is presented. Referred [31] |
| <https://doi.org/10.1002/mop.33666> | Steerable composite right–left-hand-based printed antenna circuitry for 5G applications | In this article, the proposed antenna structure is designed for modern wireless  communication systems. The antenna structure is consistent with 11‐unit cells  of composite right–left‐hand (CRLH) inclusions mounted on an FR4 substrate.  Therefore, the antenna size is miniaturized effectively  Referred [32] |
